# Supplementary material for: cyCombine allows for robust integration of single-cell cytometry datasets within and across technologies
Source: Nat Commun. 2022 Mar 31;13:1698. doi: 10.1038/s41467-022-29383-5 (PMC8971492; doi:10.1038/s41467-022-29383-5)
Supplement: Supplementary file 1 — Supplementary Information [file 41467_2022_29383_MOESM1_ESM.pdf]

Supplementary Figures

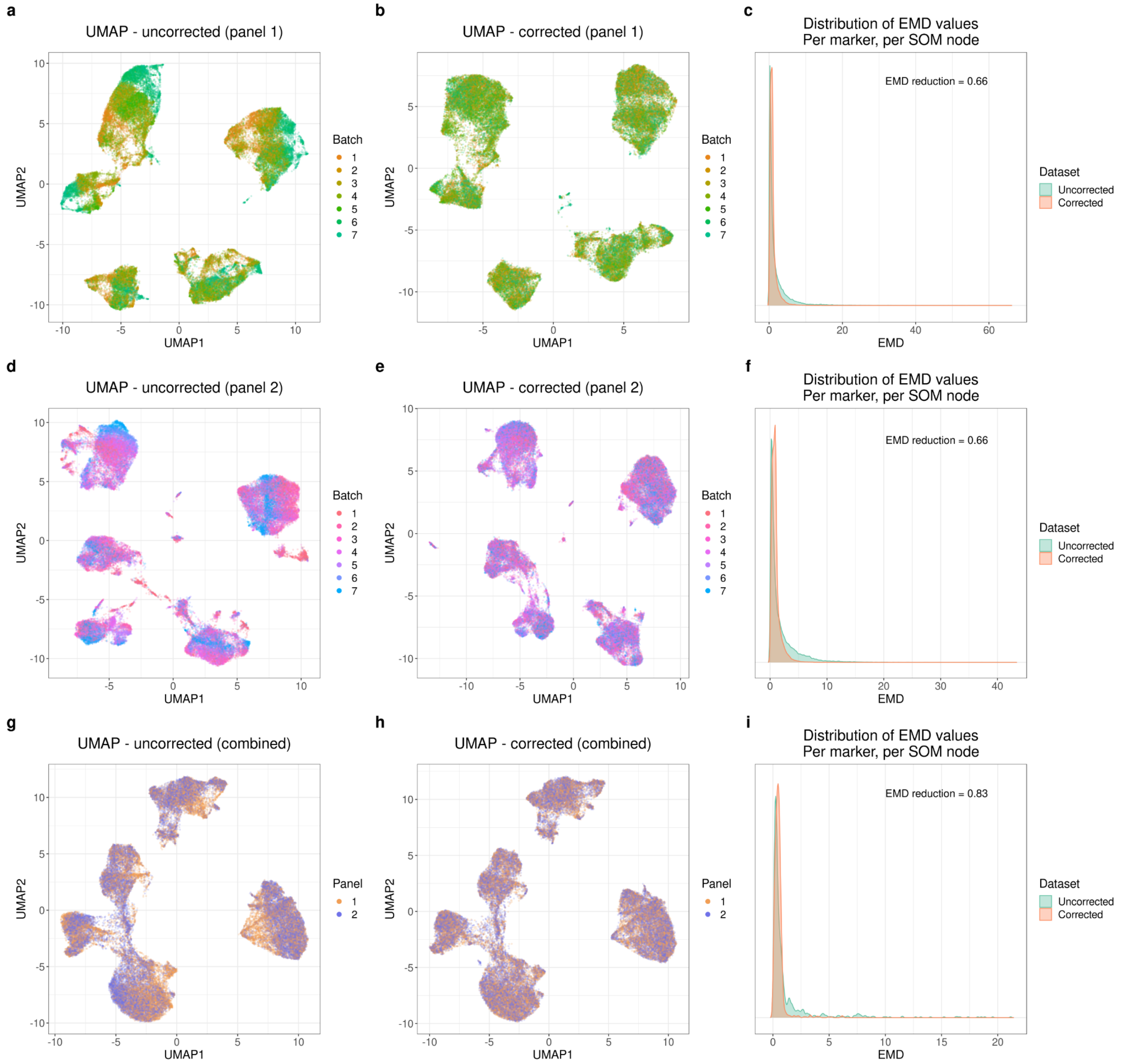

Supplementary Figure 1. Batch correction of the chronic lymphocytic leukemia (CLL) and healthy donor (HD) CyTOF dataset. **a-b**: UMAPs based on 36 markers for the panel 1 data before and after batch correction, generated using equal sampling of each batch to a total of 50,000 cells. **c**: Earth mover's distance (EMD) density plots for uncorrected and corrected data, per marker, per self-organizing map (SOM) node. The EMD reduction was 0.66 and the MAD score was 0.02. **d-f**: Same as **a-c** but for panel 2 and its 34 markers. The EMD reduction was 0.66 and the MAD score was 0.02. **g-i**: Same as **a-c** but for the co-batch correction of panels 1 and 2 and the 15 overlapping markers, using 25,000 cells per panel. The EMD reduction was 0.83 and the MAD score was 0.01.

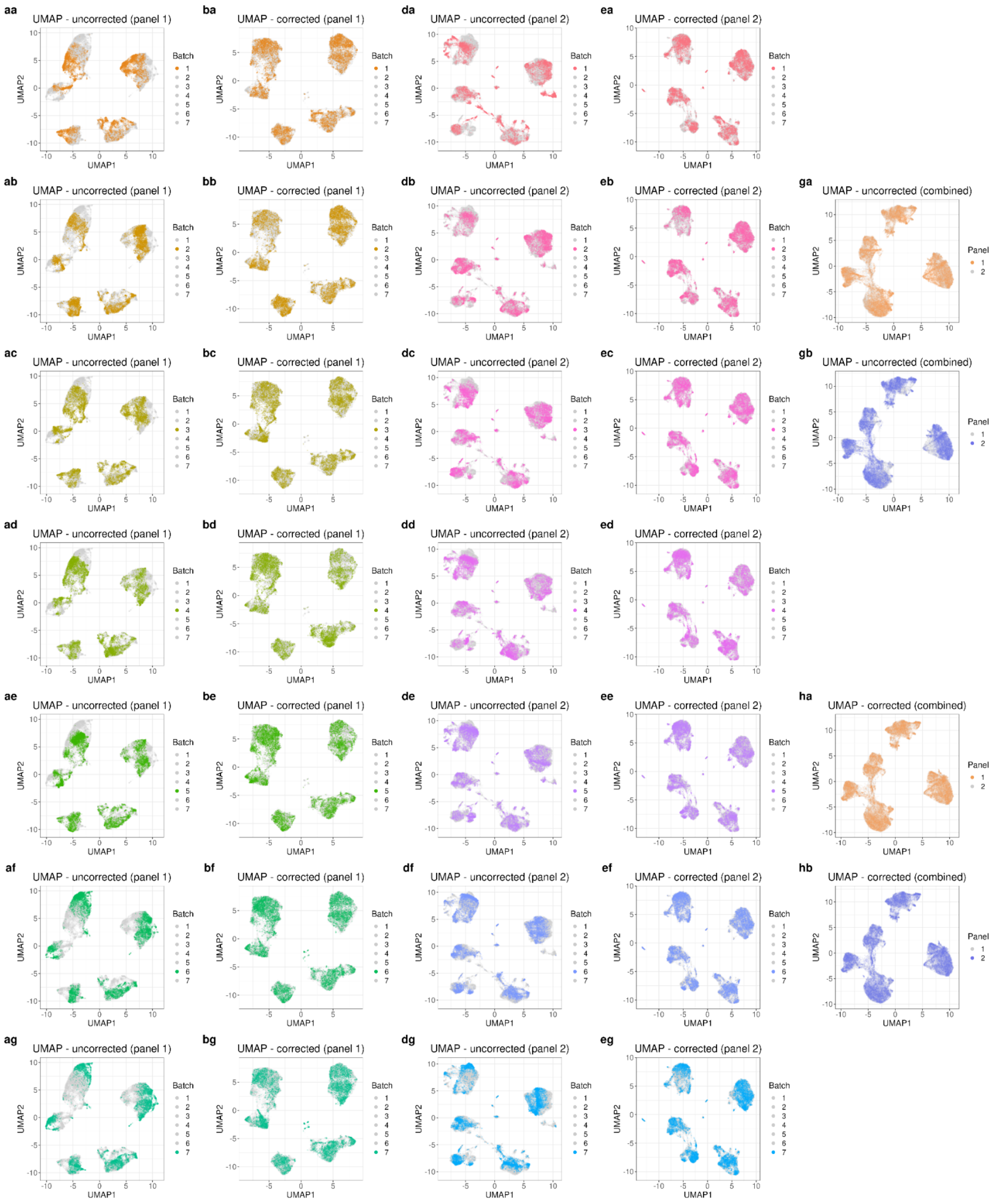

Supplementary Figure 2. Batch correction of the chronic lymphocytic leukemia (CLL) and healthy donor (HD) CyTOF dataset. **aa-bg**: UMAPs based on 36 markers for the panel 1 data before (aa-ag) and after (ba-bg) batch correction, generated using equal sampling of each batch to a total of 50,000 cells. Each plot contains cells from a single batch in color and the remaining batches in grey. **da-eg**: Same as **aa-bg** but for panel 2 and its 34 markers. **ga-hb**: Same as **aa-bg** but for the co-batch correction of panels 1 and 2 and the 15 overlapping markers, using 25,000 cells per panel.

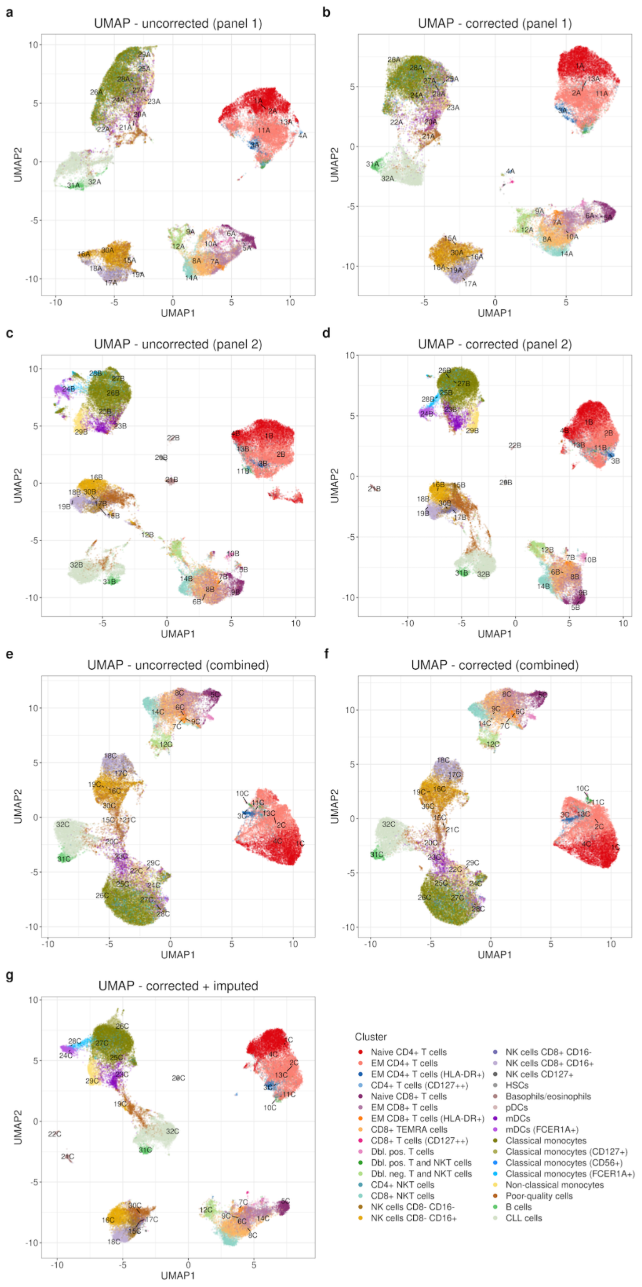

Supplementary Figure 3. Batch correction of the chronic lymphocytic leukemia (CLL) and healthy donor (HD) CyTOF dataset. All plots are colored by the labels assigned after clustering of the corrected and imputed dataset using the 23 lineage markers. The plots include B, CLL, and poor-quality cells. **a-b**: UMAPs based on 36 markers for the panel 1 data before and after batch correction, generated using equal sampling of each batch to a total of 50,000 cells. 32 cells (one randomly selected cell per cluster label) have been traced between the two plots (marked with 1A-32A). **c-d**: Same as **a-b** but for panel 2 and its 34 markers. Traced cells are marked with 1B-32B. **e-f**: Same as **a-b** but for the co-batch correction of panels 1 and 2 and the 15 overlapping markers, using 25,000 cells per panel. Traced cells are marked with 1C-32C. **g**: UMAP of the same 50,000 cells as in **e-f**, but after panel merging through imputation using all 55 markers. The traced cells are marked with 1C-32C.

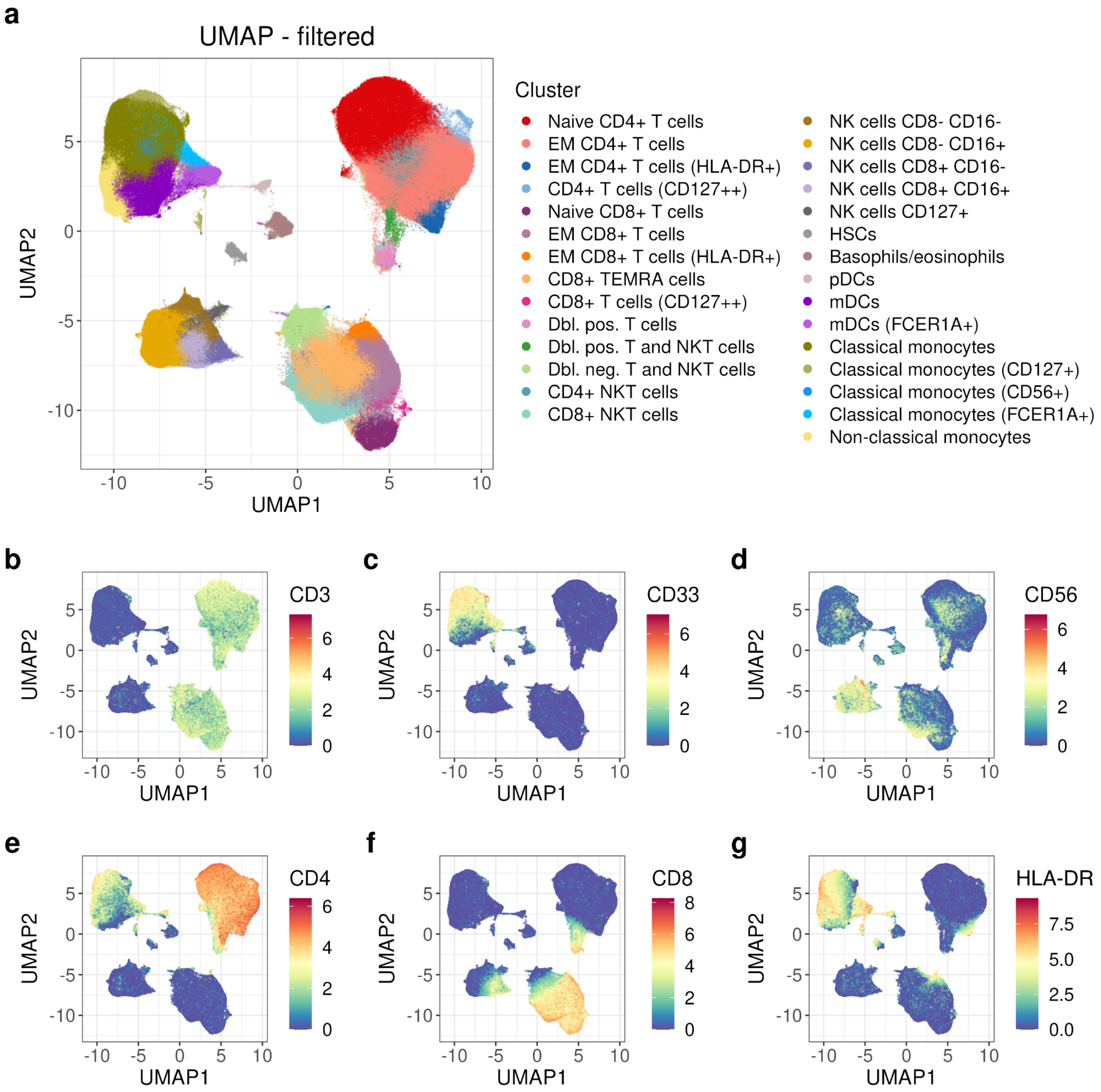

Supplementary Figure 4. Clustering results for the chronic lymphocytic leukemia (CLL) and healthy donor (HD) CyTOF dataset. **a**: UMAP for up to 4,000 cells from each of the 128 samples based on expression of the 23 clustering markers after removal of B, CLL, and poor-quality cells. **b-g**: Same as in **a**, but colored by expression of CD3, CD33, CD56, CD4, CD8, and HLA-DR.

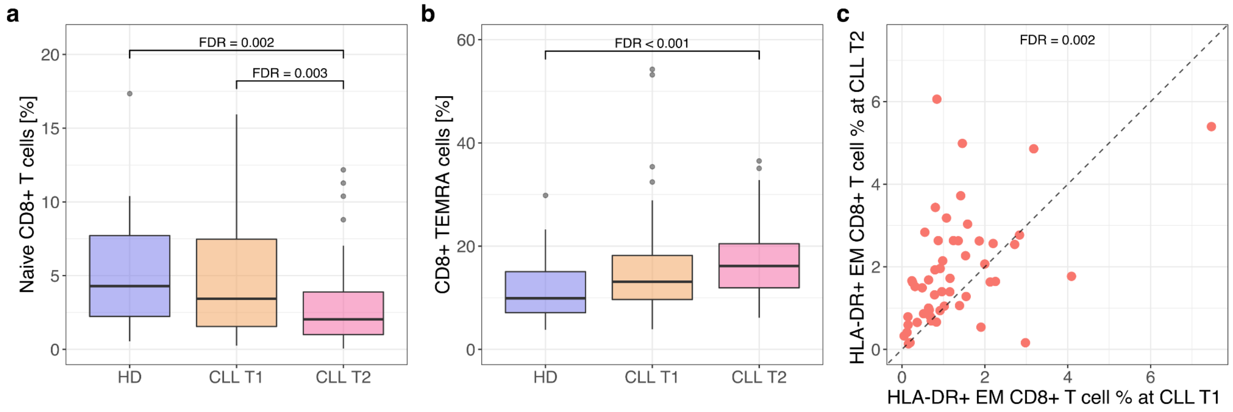

Supplementary Figure 5. Analysis of differential abundance within the T and NKT cell compartment in chronic lymphocytic leukemia (CLL) and healthy donor (HD) CyTOF data. **a-b**: Relative proportions of selected T and NKT cell populations for HD (*n* = 20), CLL time point 1 (T1) (*n* = 52), and CLL time point 2 (T2) (*n* = 56) samples: Naive CD8+ T cells and CD8+ terminally differentiated effector memory (TEMRA) cells. FDR values provided for significant comparisons. The box plots show the medians (solid line in boxes), 25th and 75th percentiles as lower and upper hinges of the boxes, and whiskers extend to the furthest data point within 1.5 * interquartile range from the hinges. Data points beyond this threshold are shown as circles. **c**: Scatter plot for the relative proportions of the paired (*n* = 52) CLL T1 and T2 patients in HLA-DR+ effector memory (EM) CD8+ T cells. False discovery rate (FDR) values provided for significant comparisons between CLL T2 vs. T1.

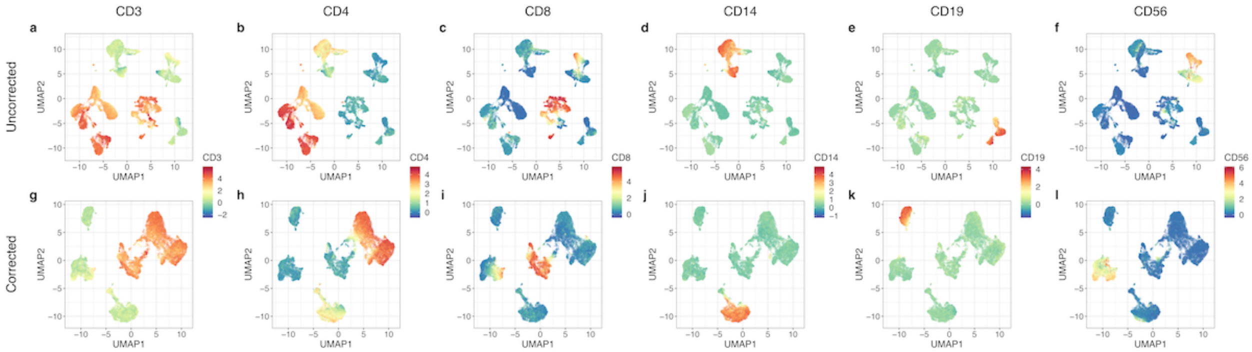

Supplementary Figure 6. Cross-platform data integration. **a-f**: UMAP plot for the uncorrected dataset consisting of 6,776 cells from each of the CITE-seq, CyTOF, and spectral flow cytometry (SFC) datasets faceted by technology and colored by major immune lineage markers; CD3, CD4, CD8, CD14, CD19, and CD56, respectively. **g-l**: Same as in **a-f**, but for the corrected dataset.

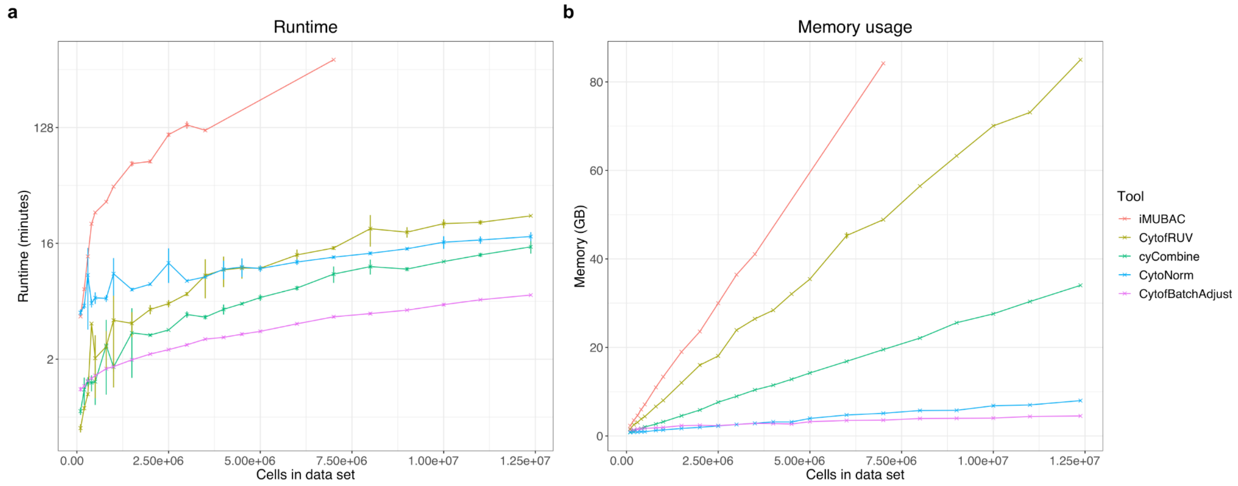

Supplementary Figure 7. Computational requirements of cyCombine and four other batch correction tools for a dataset with 38 markers and seven batches. **a**: Runtime in minutes (notice log scale on y axis) and **b**: Memory usage in GB. 40 cores and 100 gb memory were used for the system. In both panels, data are presented as mean values +/- SD based on *n* = 3 runs for the given combination of dataset size and tool.

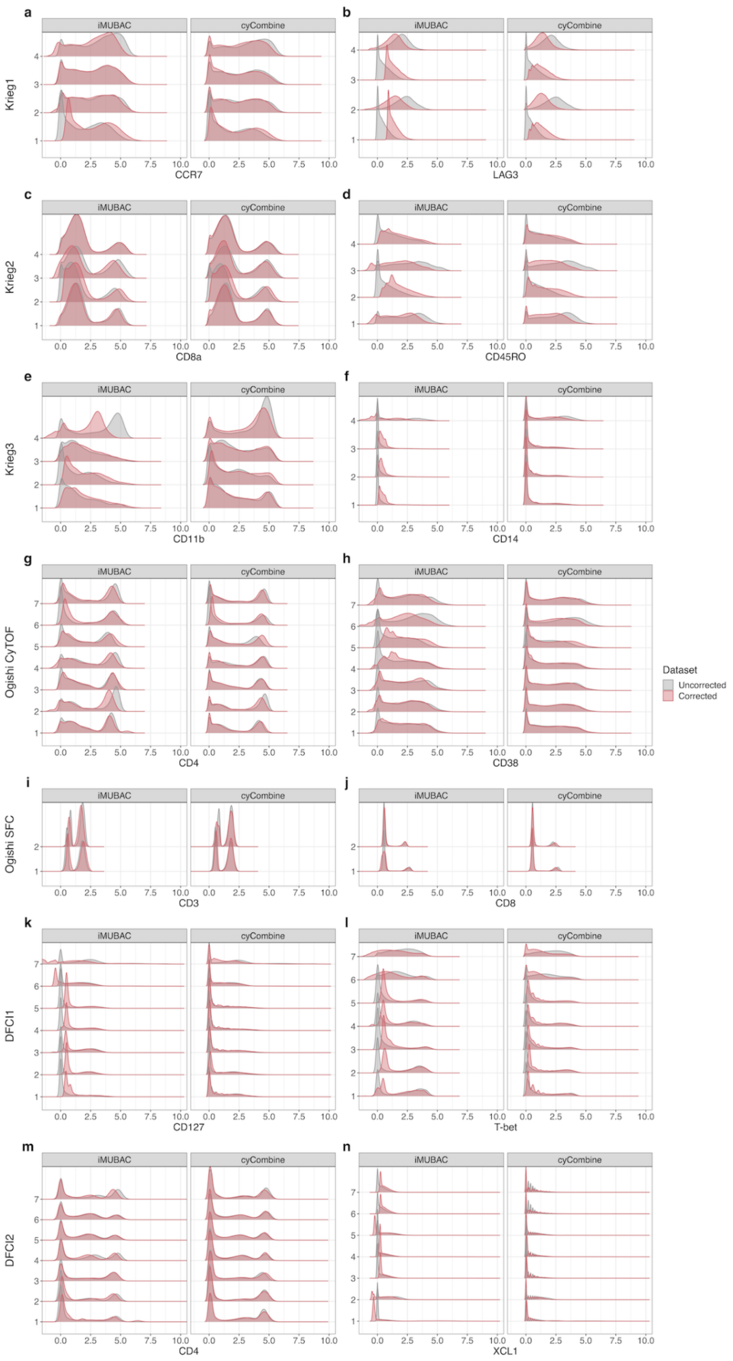

Supplementary Figure 8. Density plots for selected markers in the datasets without technical replicates before and after batch correction using iMUBAC (subsampled healthy donor samples) and cyCombine (full datasets), respectively. Markers were specifically selected to provide insights into where the tools differ in performance. Batches are indicated on the y axes. **a-b**: Expression of CCR7 and LAG3 in the Krieg1 data. **c-d**: Expression of CD8a and CD45RO in the Krieg2 data. **e-f**: Expression of CD11b and CD14 in the Krieg3 data. **g-h**: Expression of CD4 and CD38 in the Ogishi$_{CyTOF}$ data. **i-j**: Expression of CD3 and CD8 in the Ogishi$_{SFC}$ data. **k-l**: Expression of CD127 and T-bet in the DFCI1 data. **m-n**: Expression of CD4 and XCL1 in the DFCI2 data.

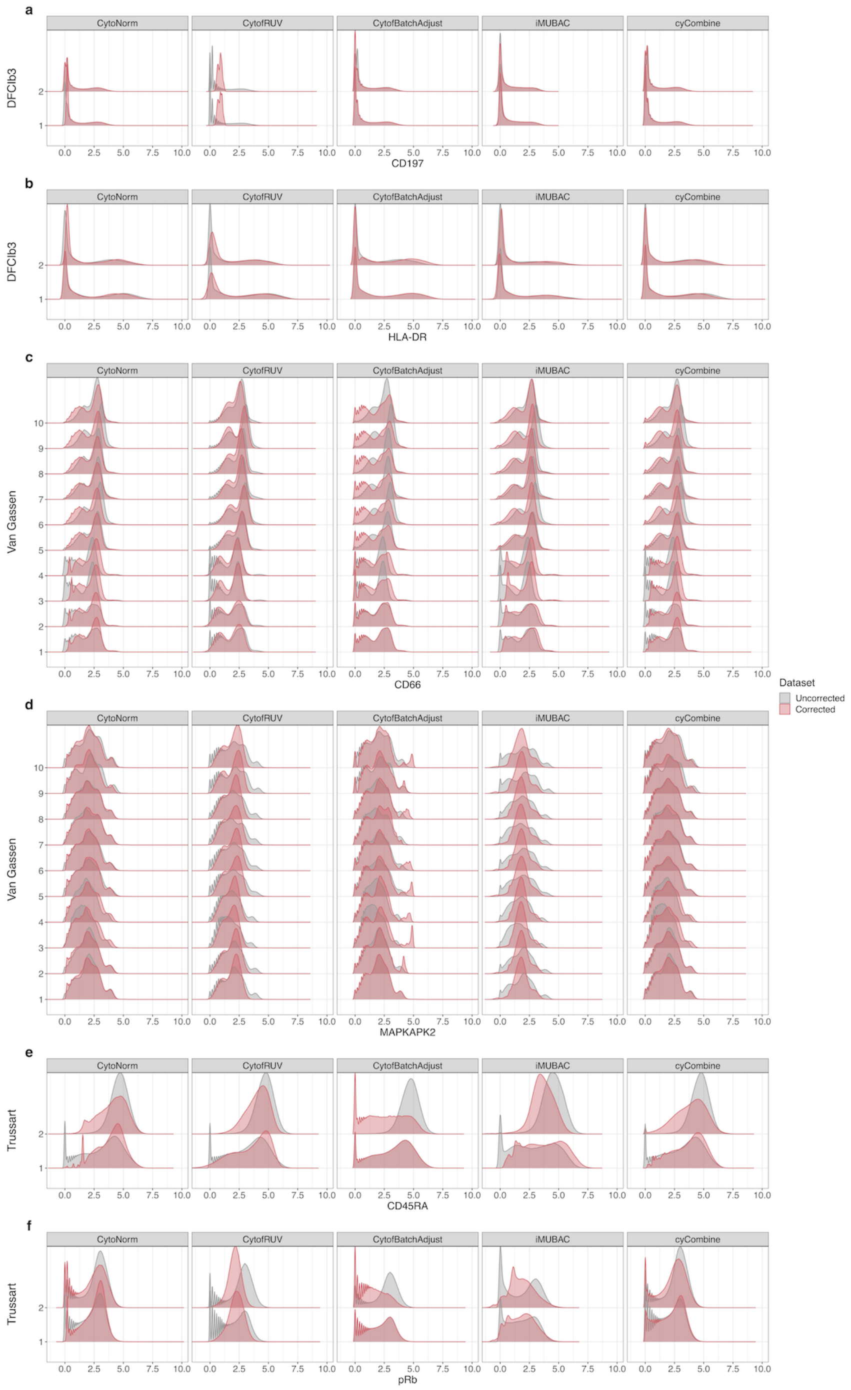

Supplementary Figure 9. Density plots for selected markers in the datasets with technical replicates before and after batch correction using CytoNorm (without samples used as technical replicates), CytofRUV (full datasets), CytofBatchAdjust (full datasets), iMUBAC (subsampled healthy donor samples), and cyCombine (full datasets), respectively. Markers were specifically selected to provide insights into where the tools differ in performance. Batches are indicated on the y axes. **a-b**: Expression of CD197 and HLA-DR in the DFCIb3 data. **c-d**: Expression of CD66 and MAPKAPK2 in the Van Gassen data. **e-f**: Expression of CD45RA and pRb in the Trussart data.

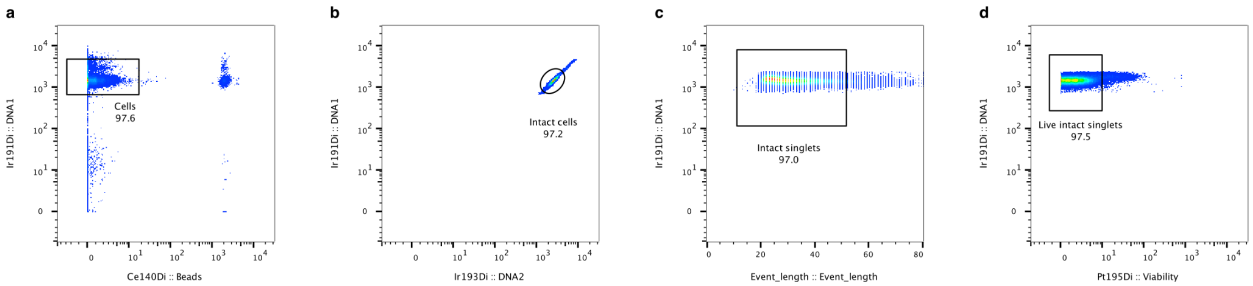

Supplementary Figure 10. Manual pre-gating strategy for the chronic lymphocytic leukemia (CLL) and healthy donor (HD) CyTOF dataset, exemplified by a single HD sample. Pre-gating was carried out in four steps using FlowJo version 10 (Tree Star Inc). The percentage of events within gates is indicated relative to the number of “parent” events. **a**: Gating of cells based on the measurements for Beads and DNA1. **b**: Gating of intact cells based on the measurements for DNA2 and DNA1. **c**: Gating of intact singlets based on the measurements for Event length and DNA1. **d**: Gating of live intact singlets based on the measurements for Viability and DNA1.

Supplementary Discussion

Panel merging

Previous work by Abdelaal et al. (2019)[1] suggested approaches for 1) designing panels with an optimal overlap for imputation and 2) imputation based on the median expressions of markers in k-Nearest Neighbors (kNN). Their tool, CyTOFmerge[1] was developed for designing experiments that allow for integration of multi-panel data, and not for merging pre-designed panels. As such, the tool handles low variance markers quite well, but intuitively, median-based imputation is not optimal for multimodal distributions. Lee et al.[2] presented a flow cytometry method for merging purposes, but this works only for two files at a time, relies on domain knowledge, and has only been tested on lymphocyte data. CytoBackBone[3] offers a solution more similar to the cyCombine panel merging module. For combining and integrating datasets, approaches like QFMatch[4], SIC[5], and MetaCyto[6] have also been presented. However, these are focused on combining the results of complete analyses and not on allowing truly integrated analysis from start to end. A thorough evaluation and discussion of the panel merging module of cyCombine and the existing alternatives is included in a vignette at https://biosurf.org/cyCombine.

In the analysis of the chronic lymphocytic leukemia (CLL) data in this article, we rely on panel merging for clustering and visualization of data generated from the same samples using two different panels. To illustrate the impact of the panel merging step on the information content in the samples, **Supplementary Figure 3** shows the final clusters on UMAPs generated at different analysis stages. In this figure, it can be seen that some of the clusters obtained with the final, integrated dataset are not well-separated when considering only the markers available in a single panel. One example is the clear combination of the myeloid clusters in **Supplementary Figure 3a-b**, which is caused by the lack of multiple important myeloid markers, such as CD123, CD11b, and CD1c, in panel 1. Similarly, in **Supplementary Figure 3c-d**, we observe a combination of CD8+ T cell types of the effector memory (EM) and terminally differentiated effector memory (TEMRA) compartments, most likely caused by the lack of CD45RO in panel 2. Both of these separation failures are visible in the co-batch corrected set with only 15 markers, shown in **Supplementary Figure 3e-f**. However, when considering the final, integrated set in **Supplementary Figure 3g**, we observe both a clear separation of the myeloid populations - similar to what is observed for panel 2 alone - and a good definition of CD8+ T cell subtypes - as in panel 1. This shows that while there are discrepancies between the clusters that can be obtained with and without the use of panel merging, the final clusters have real biological measurements supporting their existence.

Analysis of CLL data

Given that CLL can severely affect bone-marrow production of immune and hematopoietic cells[7,8], immune dysfunction in CLL is to be expected. The focus of our analysis was to identify features of the immune system that differentiate CLL patients from healthy donors (HDs) and patients sampled at different times relative to treatment initiation. After integrating the two panels of the CLL dataset, we compared the overall frequency for each of the 29 populations in cells originating from panel 1 and panel 2, respectively. We observed a very strong Pearson

correlation of 0.9996 making it reasonable to assume that any merged sample may be considered as a single combined sample for differential abundance analysis.

In this analysis, we found that, compared to HDs, close-to-treatment time point 2 (T2) CLL patients had higher amounts of T and NKT cells as well as hematopoietic stem cells (HSCs) (CD34+) (**Figure 2**). For HD vs. CLL time point 1 (T1), similar patterns were observed, but the difference in HSC frequencies was not statistically significant. However, when comparing CLL T2 vs. CLL T1 while accounting for the paired samples, the HSCs were significantly more abundant (logFC = 0.7) at CLL T2. Additionally, the most dramatic difference for HD vs. CLL T2 was also the HSCs (logFC = 1.2).

It is currently debated whether the absolute T cell counts in CLL are higher[9–11] or lower[12] than those found in HDs. Our results show a higher proportion of T and NKT cells in CLL patients, compared to HD, which is in line with the majority of the published works. Furthermore, the effects of CLL on the T cell compartment are also widely discussed[9–14], and in order to investigate the T and NKT cell compartment more deeply, we considered the populations as "daughters" of their overall type, meaning that proportions were relative to the parent set of T and NKT clusters. This was done to account for the compositional nature of the data, which means that changes in overall cell type proportions can mask population-specific differences (when one population increases in frequency, the sum of frequencies of the rest of the populations will go down).

Within the group of T and NKT cells, we found that when comparing HD vs. CLL T1, the CLL samples had lower proportions of HLA-DR+ effector memory (EM) CD4+ T cells, and for CLL T2 vs. HD, we observed significantly lower levels of naive CD8+ T cells (**Supplementary Figure 5a**) and higher abundances of CD8+ terminally differentiated effector memory (TEMRA) cells in the CLL samples (**Supplementary Figure 5b**). Previous studies have also reported a general decrease in the naive T cell compartment for CLL patients[12,13]. Skews towards CD8+ EM T and TEMRA cells among CLL patients have also been reported[12,15], as well as a general increase in antigen-experienced[11] or memory T cells[13], as seen here, indicative of a low output of naive T cells.

Within the CLL patients, additional significant changes were detected. Overall, the two populations of HLA-DR+ EM CD8+ and CD4+ T cells constituted larger proportions of the total T and NKT cell compartment at T2 (**Figure 2f** and **Supplementary Figure 5c**), whereas the naive CD8+ T cells were less abundant at T2.

Elston et al. (2019)[13] associated a subpopulation of CD4+PD-1+HLA-DR+ T cells to progression in CLL, and specifically mentioned that this is most frequent in the EM compartment. PD-1+ expression patterns have also been discussed in relation to replicative senescence, which is associated with more aggressive disease in CLL patients[12]. In our cohort, we found PD-1 to be most highly expressed by the two HLA-DR+ EM T cell subsets, indicating that these clusters may actually encompass the PD-1+ fraction as well, supporting existing results. Within the

CD4+ cluster, there was also a significantly higher median expression of PD-1 in CLL T2 samples compared to HDs (logFC = 0.47) (**Figure 2g**).

Taken together, our analysis of CLL patients shows that applying cyCombine to a multi-batch dataset enables co-analysis leading to the identification of characteristics commonly ascribed to the CLL immunophenotype, as well as novel cellular phenotypes only detectable when combining large panels.

Benchmarking

For data integration purposes, the total size of datasets may be very large. This means that that ideal batch correction tool has good scaling in terms of runtime and memory usage. We investigated this for the four tools we tested and compared to cyCombine (**Supplementary Figure 7**). All the tools scaled approximately linearly in their memory usage, with cyCombine showing medium usage. In terms of runtime, cyCombine scales approximately linearly and three of the other included tools have similar runtime scaling.

For the benchmark test, we aimed to test all tools on as many of the datasets used in the publications as possible. For several of those (Van Gassen and Trussart), and for the DFCIb3 dataset, we note that many samples are technical replicates, which is perhaps not an ideal test for batch correction methods, as biological variance is expected to be very close to zero. This means that all the variance between those batches would be subject to removal, which is essentially only half the challenge (the other half being conservation of biological variance). We also noted that some of the included tools are not geared for correction of samples with non-identical panels. The DFCI dataset is composed of two different panels, and HLA-DR was labeled with different metal isotopes in the two panel. Because the tools read samples directly from the FCS format, it is hard to process such cases and the issue must be addressed by either altering the actual FCS files, or editing the source code of the tools. cyCombine is more flexible since it works on R data.frames, which are straightforward to edit.

Supplementary References

1. Abdelaal, T. *et al.* CyTOFmerge: Integrating mass cytometry data across multiple panels. *Bioinformatics* (2019) doi:10.1093/bioinformatics/btz180.
2. Lee, G., Finn, W. & Scott, C. Statistical file matching of flow cytometry data. *J. Biomed. Inform.* **44**, 663–676 (2011).
3. Leite Pereira, A., Lambotte, O., Le Grand, R., Cosma, A. & Tchitchek, N. CytoBackBone: an algorithm for merging of phenotypic information from different cytometric profiles. *Bioinformatics* **35**, 4187–4189 (2019).
4. Orlova, D. Y. *et al.* QFMatch: multidimensional flow and mass cytometry samples alignment. *Sci. Rep.* **8**, 3291 (2018).
5. Meehan, S. *et al.* Automated subset identification and characterization pipeline for multidimensional flow and mass cytometry data clustering and visualization. *Commun. Biol.* **2**, 229 (2019).
6. Hu, Z. *et al.* MetaCyto: A Tool for Automated Meta-analysis of Mass and Flow Cytometry Data. *Cell Rep.* **24**, 1377–1388 (2018).
7. Arruga, F. *et al.* Immune response dysfunction in chronic lymphocytic leukemia: dissecting molecular mechanisms and microenvironmental conditions. *Int. J. Mol. Sci.* **21**, (2020).
8. Purroy, N. & Wu, C. J. Coevolution of leukemia and host immune cells in chronic lymphocytic leukemia. *Cold Spring Harb. Perspect. Med.* **7**, (2017).
9. Scrivener, S., Goddard, R. V., Kaminski, E. R. & Prentice, A. G. Abnormal T-cell function in B-cell chronic lymphocytic leukaemia. *Leuk. Lymphoma* **44**, 383–389 (2003).
10. Gonzalez-Rodriguez, A. P. *et al.* Prognostic significance of CD8 and CD4 T cells in chronic lymphocytic leukemia. *Leuk. Lymphoma* **51**, 1829–1836 (2010).
11. Palma, M. *et al.* T cells in chronic lymphocytic leukemia display dysregulated expression of immune checkpoints and activation markers. *Haematologica* **102**, 562–572 (2017).
12. Nunes, C. *et al.* Expansion of a CD8(+)PD-1(+) replicative senescence phenotype in early stage CLL patients is associated with inverted CD4:CD8 ratios and disease progression. *Clin. Cancer Res.* **18**, 678–687 (2012).
13. Elston, L. *et al.* Increased frequency of CD4+ PD-1+ HLA-DR+ T cells is associated with disease progression in CLL. *Br. J. Haematol.* **188**, 872–880 (2020).
14. D'Arena, G. *et al.* Regulatory T-cell number is increased in chronic lymphocytic leukemia patients and correlates with progressive disease. *Leuk. Res.* **35**, 363–368 (2011).
15. Riches, J. C. *et al.* T cells from CLL patients exhibit features of T-cell exhaustion but retain capacity for cytokine production. *Blood* **121**, 1612–1621 (2013).
