# Supplementary material for: cyCombine allows for robust integration of single-cell cytometry datasets within and across technologies
Source: Nat Commun. 2022 Mar 31;13:1698. doi: 10.1038/s41467-022-29383-5 (PMC8971492; doi:10.1038/s41467-022-29383-5)
Supplement: Supplementary file 2 — Reporting Summary [file 41467_2022_29383_MOESM2_ESM.pdf]

nature research

Corresponding author(s): Lars Rønn Olsen

Last updated by author(s): 2022/03/02

Reporting Summary

Nature Research wishes to improve the reproducibility of the work that we publish. This form provides structure for consistency and transparency in reporting. For further information on Nature Research policies, see our Editorial Policies and the Editorial Policy Checklist.

Statistics

For all statistical analyses, confirm that the following items are present in the figure legend, table legend, main text, or Methods section.

n/a	Confirmed	
☐	☒	The exact sample size (*n*) for each experimental group/condition, given as a discrete number and unit of measurement
☐	☒	A statement on whether measurements were taken from distinct samples or whether the same sample was measured repeatedly
☐	☒	The statistical test(s) used AND whether they are one- or two-sided<br>*Only common tests should be described solely by name; describe more complex techniques in the Methods section.*
☐	☒	A description of all covariates tested
☐	☒	A description of any assumptions or corrections, such as tests of normality and adjustment for multiple comparisons
☐	☒	A full description of the statistical parameters including central tendency (e.g. means) or other basic estimates (e.g. regression coefficient) AND variation (e.g. standard deviation) or associated estimates of uncertainty (e.g. confidence intervals)
☐	☒	For null hypothesis testing, the test statistic (e.g. *F*, *t*, *r*) with confidence intervals, effect sizes, degrees of freedom and *P* value noted<br>*Give P values as exact values whenever suitable.*
☒	☐	For Bayesian analysis, information on the choice of priors and Markov chain Monte Carlo settings
☒	☐	For hierarchical and complex designs, identification of the appropriate level for tests and full reporting of outcomes
☐	☒	Estimates of effect sizes (e.g. Cohen's *d*, Pearson's *r*), indicating how they were calculated

Our web collection on statistics for biologists contains articles on many of the points above.

Software and code

Policy information about availability of computer code

Data collection	Fluidigm CyTOF Software v7.0 was used for collection of mass cytometry data.
Data analysis	Custom code available via https://github.com/biosurf/cyCombine, and/or in the use case vignettes. Reported versions: R v. 4.0.0, kohonen v. 3.0.10, ConsensusClusterPlus v. 1.54.0, sva v. 3.38.0, ggplot2 v. 3.3.3, uwot v. 0.1.9, ggridges v. 0.5.2, patchwork v. 1.1.1, Seurat v. 4.0.0, CATALYST v. 1.12.2, premessa v. 0.2.6, iMUBAC v. 0.1.1, CytofRUV v. 0.1, CytoNorm v. 0.0.5

For manuscripts utilizing custom algorithms or software that are central to the research but not yet described in published literature, software must be made available to editors and reviewers. We strongly encourage code deposition in a community repository (e.g. GitHub). See the Nature Research guidelines for submitting code & software for further information.

Data

Policy information about availability of data

All manuscripts must include a data availability statement. This statement should provide the following information, where applicable:
- Accession codes, unique identifiers, or web links for publicly available datasets
- A list of figures that have associated raw data
- A description of any restrictions on data availability

The DFCI CyTOF data generated in this study have been deposited in the FlowRepository database under accession code FR-FCM-Z52G [https://flowrepository.org/id/FR-FCM-Z52G].
The HIMC CyTOF data used in this study are available in the FlowRepository database under accession code FR-FCM-ZYAJ [https://flowrepository.org/id/FR-FCM-ZYAJ]. The Park flow data used in this study are available in the FlowRepository database under accession code FR-FCM-Z2QV [https://flowrepository.org/id/FR-FCM-Z2QV]. The van Gassen CyTOF data used in this study are available in the FlowRepository database under accession code FR-FCM-Z247 [https://flowrepository.org/id/FR-FCM-Z247]. The Trussart CyTOF data used in this study are available in the FlowRepository database under accession code FR-FCM-Z2L2

[https://flowrepository.org/id/FR-FCM-Z2L2]. The Krieg CyTOF data used in this study are available in the FlowRepository database under accession code FR-FCM-ZY34 [https://flowrepository.org/id/FR-FCM-ZY34]. The Ogishi CyTOF data used in this study are available in the FlowRepository database under accession code FR-FCM-Z3YK [https://flowrepository.org/id/FR-FCM-Z3YK]. The Ogishi flow cytometry data used in this study are available in the FlowRepository database under accession code FR-FCM-Z3YL [https://flowrepository.org/id/FR-FCM-Z3YL]. The CITE-seq data used in this study are available from the 10X genomics website [https://support.10xgenomics.com/single-cell-gene-expression/datasets/3.0.0/pbmc_10k_protein_v3].

Field-specific reporting

Please select the one below that is the best fit for your research. If you are not sure, read the appropriate sections before making your selection.

- [x] Life sciences
- [ ] Behavioural & social sciences
- [ ] Ecological, evolutionary & environmental sciences

For a reference copy of the document with all sections, see nature.com/documents/nr-reporting-summary-flat.pdf

Life sciences study design

All studies must disclose on these points even when the disclosure is negative.

Sample size	We selected a large cohort of primary patient material, that reflected the broad natural history of CLL. This sample size was selected based on the availability of samples within the CLL biobank. This study represents the largest mass cytometric assessment of CLL reported.
Data exclusions	Poor quality samples were excluded as per the methods
Replication	We had access to limited numbers of primary patient material and replication of cytof runs was not possible. However, we performed quality control assessment to allow for standardisation across batches and runs, which we assessed during the analysis of the results.
Randomization	Not relevant to this study as this study was not a trial related
Blinding	Blinding was not possible but all samples were processed and analysed identically to avoid any unintentional bias introduced

Reporting for specific materials, systems and methods

We require information from authors about some types of materials, experimental systems and methods used in many studies. Here, indicate whether each material, system or method listed is relevant to your study. If you are not sure if a list item applies to your research, read the appropriate section before selecting a response.

Materials & experimental systems

n/a	Involved in the study	
[ ]	[x]	Antibodies
[x]	[ ]	Eukaryotic cell lines
[x]	[ ]	Palaeontology and archaeology
[x]	[ ]	Animals and other organisms
[ ]	[x]	Human research participants
[x]	[ ]	Clinical data
[x]	[ ]	Dual use research of concern

Methods

n/a	Involved in the study	
[x]	[ ]	ChIP-seq
[x]	[ ]	Flow cytometry
[x]	[ ]	MRI-based neuroimaging

Antibodies

Antibodies used

CD20, 2H7, 113In, BioLegend
CD3, UCHT1, 115In, BioLegend
CD27, O323, 141Pr, BioLegend
CD45RA, HI100, 142Nd, BioLegend
CD279 (PD1), EH12.2H7, 143Nd, BioLegend CD5, UCHT2, 144Nd, BioLegend
CD19, HIB19, 145Nd, BioLegend
CD14, M5E2, 146Nd, BioLegend
CD45RO, UCHL1, 147Sm, BioLegend GZMA, CB9, 148Nd, BioLegend
GZMK, GM26E7, 149Sm, BioLegend FCRL6, 7B7, 150Nd, BioLegend
CD355 (CRTAM), Cr24.1, 151Eu, BioLegendCD152 (CTLA4), L3D10, 152Sm, BioLegend
CD69, FN50, 153Eu, BioLegend
CD33, WM53, 154Sm, BioLegend
CD4, RPA-T4, 155Gd, BioLegend
CD337 (NCR3), P30-15, 156Gd, BioLegend CD8, RPA-T8, 158Gd, BioLegend
CD197 (CCR7), G043H7, 159Tb, BioLegendLAG-3, 874501, 161Dy, R&D CD56, NCAM16.2, 162Dy, BD

CD137 (4-1BB), 4B4-1, 163Dy, BioLegend
CD161 (KLRB1), HP-3G10, 164Dy, BioLegend FoxP3, PCH101, 165Ho, eBioscience
CD80, 2D10, 166Er, BioLegend
CD270 (HVEM), 122, 167Er, BioLegend CD275 (ICOSL, B7-H2, 136726, 168Er, R&D CD134 (OX40), Ber-ACT35, 169Tm, BioLegendCD278 (ICOS), C398.4A, 170Er, BioLegend
CD127, RDR5, 171Yb, eBioscience KLRG1, 2F1/KLRG1, 172Yb, BioLegendCD25, M-A251, 173Yb, BioLegend HLA-DR, L243, 174Yb , BioLegend
T-Bet, 4B10, 175Lu, BioLegend XCL1, Polyclonal, 176Yb, R&D CD20, 2H7, 113In, BioLegend
CD3, UCHT1, 115In, BioLegend CD45RA, HI100, 142Nd, BioLegend CD1c, L161, 143Nd, BioLegend CD5, UCHT2, 144Nd, BioLegend CD19, HIB19, 145Nd, BioLegend CD14, M5E2, 146Nd, BioLegend HLA-DR, L243, 147Sm,
BioLegend
CD1d, 51.1, 149Sm, BioLegend
CD11c, Bu15, 150Nd, BioLegend
CD123, 6H6, 151Eu, BioLegend
JAK1, 413104, 153Eu, R&D
CD33, WM53, 154Sm, BioLegend
CD4, RPA-T4, 155Gd, BioLegend
CD16, 3G8, 157Gd, BioLegend
CD8, RPA-T8, 158Gd, BioLegend
CD197, G043H7, 159Tb, BioLegend IFNG, 4S.B3, 160Gd, BioLegend
CD74, LN2, 161Dy, BioLegend
CD56, NCAM16.2, 162Dy, BD
DR3 (TRAMP), JD3, 163Dy, BioLegend CD161 (KLRB1), HP-3G10, 164Dy, BioLegend
FoxP3, PCH101, 165Ho, eBioscience CD34, 581, 166Er, BioLegend
IL23A, HLT2736, 167Er, BioLegend SMAD2, 376520, 168Er, R&D
CD11b, M1/70, 169Tm, BioLegend CD184 (CXCR4), 12G5, 171Yb, BioLegend TGFBR2, 16H2L4, 172Yb, Invitrogen FCeR1a, AER-37, 173Yb, BioLegend TGFB1, TW4-2F8 , 174Yb , BioLegend CD54 (ICAM1), HA58, 175Lu, BioLegendXCL1, 109001, 176Yb, R&D IL1RA, 40007, 209Bi, R&D

Validation

The antibodies used for CyTOF staining were selected as antibody clones identified by the manufacturers as being validated for flow cytometry use. Antibodies were labeled and validated by the Harvard Medical Area (HMA) CyTOF Antibody Resource and Core. All labeled antibodies were validated for protein concentrations using a NanoDrop-Lite instrument (Thermo Fisher Scientific, Waltham, MA) and for cell staining activity as well as appropriate immune cell type distribution.

Human research participants

Policy information about studies involving human research participants

Population characteristics

CLL patient samples were obtained from the CLL Research Consortium (University of San Diego) following informed consent. All patients were untreated prior to sampling. For the 56 CLL patients, the mean age at diagnosis was 56.1 years (sd = 9.6 years), with healthy donors being age-matched (mean = 56.7 years, sd = 4.5 years). 20 are female, 23 are male, 13 are of unknown sex. Healthy donor samples were obtained from the Parquarello Tissue Bank at Dana Farber Cancer Institute or from commercial sources. 5 are female, 15 are male.

Recruitment

Participant samples were recruited from the CLL Research Consortium Biobank (University of San Diego). This biobank collects standardised samples from a number of US and international centres. We purposely selected untreated patients at 2 time points, in a total 56 of patients to overcome any potential bias.

Ethics oversight

Samples were collected following IRB approval from the University of California San Diego Project 171884

Note that full information on the approval of the study protocol must also be provided in the manuscript.
