# Supplementary material for: cyCombine allows for robust integration of single-cell cytometry datasets within and across technologies
Source: Nat Commun. 2022 Mar 31;13:1698. doi: 10.1038/s41467-022-29383-5 (PMC8971492; doi:10.1038/s41467-022-29383-5)
Supplement: Supplementary file 4 — Description of Additional Supplementary Files [file 41467_2022_29383_MOESM4_ESM.pdf]

Description of Additional Supplementary Files

File Name: Supplementary Data 1
Description: CyTOF panels for the CLL and HD dataset.
